# Increases in HPV-16/18 antibody avidity and HPV-specific memory B-cell response in mid-adult aged men post-dose three of the quadrivalent HPV vaccine

**DOI:** 10.1016/j.vaccine.2021.07.069

**Published:** 2021-08-06

**Authors:** Cheryl N. Miller, Troy J. Kemp, Martha Abrahamsen, Kimberly Isaacs-Soriano, Kim Dunham, Bradley Sirak, Yuanji Pan, Eduardo Lazcano-Ponce, Jorge Salmeron, Ligia A. Pinto, Anna R. Giuliano

**Affiliations:** a Vaccine, Immunity, and Cancer Directorate, Leidos Biomedical Research, Inc., Frederick National Laboratory for Cancer Research, National Cancer Institute, Frederick, MD, USA; b Center for Immunization and Infection Research in Cancer (CIIRC), Moffitt Cancer Center, Tampa, FL, USA; c National Institute of Public Health, Cuernavaca, Morelos, Mexico

## Abstract

Strong quantitative and functional antibody responses to the quadrivalent human papillomavirus (HPV) vaccine were reported in mid-adult aged men, but there are limited data on the avidity of the antibody response and the memory B-cell response following vaccination. Although circulating antibodies induced by vaccination are believed to be the main mediators of protection against infection, evaluation of avidity of antibodies and memory B cell responses are critical for a better understanding of the vaccine immunogenicity mechanisms. Both the modified enzyme-linked immunosorbent assay (ELISA) and the enzyme-linked immunosorbent spot (ELISpot) assay are tools to measure the humoral and cellular immune responses post vaccination to characterize vaccine immunogenicity. The avidity of HPV-16 and HPV-18 specific IgG in the serum of mid-adult aged men (N = 126) who received three quadrivalent HPV vaccine doses was examined using a modified ELISA. HPV-16 memory B-cell responses were assessed via ELISpot at month 0 (prior to vaccination) and 1-month post-dose three of the vaccine (month 7). The quadrivalent vaccine induced an increase in HPV-16 and HPV-18 antibody avidity at month 7. HPV-18 avidity levels moderately correlated with anti-HPV-18 antibody titers, but no association was observed for HPV-16 antibody titers and avidity levels. The HPV-16-specific memory B-cell response was induced following three vaccine doses, however, no association with anti-HPV-16 antibody avidity was observed. Three doses of quadrivalent HPV vaccine increased antibody affinity maturation for HPV-16/18 and increased the frequency of anti-HPV-16 memory B-cells in mid-adult aged men.

## 1. Introduction

Human papillomavirus (HPV) is one of the most common sexually transmitted viruses, and a cause of several cancers in both men and women. HPV-16 and HPV-18 are the most common oncogenic HPV types, accounting for approximately 60% of oropharyngeal cancers, and approximately 90% of anal cancers [1]. The quadrivalent HPV vaccine, Gardasil, has been shown to be highly immunogenic and provides excellent protection against HPV-6/11/16/18 [2,3]. While HPV vaccination holds promise to reduce the incidence and mortality of HPV-related cancers, vaccine uptake, particularly in males remains low and sub-optimal in most countries. This is particularly problematic as men have a 4-fold higher incidence of oropharyngeal cancers compared to females [4], and a significantly higher prevalence of oral HPV than do women. Unlike cervical HPV infection, the prevalence of oral HPV increases with age among men with the highest oral HPV prevalence in men aged 55 to 74 years [5].

Routine vaccination in mid-adult aged men could help reduce the incidence of oropharyngeal cancers, by preventing oral HPV infection. The quadrivalent HPV vaccine is safe and effective in reducing HPV-6/11/16/18 anogenital infections in young males ages 16–26 years [6]. We previously demonstrated men ages 27–45 years mount a strong and specific antibody response comparable to levels in young males after three doses of quadrivalent HPV vaccine where clinical efficacy of the vaccine was demonstrated [7,8]. Further, the quadrivalent HPV vaccine administered to HIV-infected men was also effective at eliciting a strong specific immune response [9], a strong indication that protective immunity occurs among mid-adult aged men.

To further evaluate the quality of the antibody response post vaccination among mid-adult aged men, antibody avidity and number of antigen specific memory B-cells were measured. Circulating antibodies against HPV induced by vaccination play an essential role in protection against repeated exposures and subsequent HPV infections. The antibody levels that are induced by vaccination are often much higher than that observed during natural HPV infection and more detailed analysis of this robust immune response will uncover key factors in long-term immune protection against HPV infection [10–12]. The avidity index is a measure of the relative strength of the interaction between the antibody with the antigen complex and is a method to evaluate the quality of antibody responses and protection against disease [13]. There are limited studies investigating the role of avidity in protection against HPV infections because the HPV vaccines have demonstrated remarkably high efficacy [14]. However, avidity of HPV antibodies may be associated with the strong vaccine efficacy and protection against newly detected HPV infections observed in vaccine recipients [14,15]. Recall immune responses are rapid and produce high affinity antibodies because long lived memory B cells differentiate into the antibody producing plasma cells [16]. Long-lived plasma cells secrete affinity matured antibodies, which can mediate protection form HPV infection [17]. Here, we measured these humoral and cellular immune responses to determine quality of the immune response following three doses of the quadrivalent HPV vaccine among mid-adult aged men. Furthermore, we optimized an established ELISpot assay for the use with HPV-16 and assessed the associations between antibody avidity and memory B-cell responses in this population of men.

## 2. Material and methods

### 2.1. Participants and serum samples

#### 2.1.1. Study population

Sera were collected at months 0 and 7 from 150 men, ages 27–45 who received quadrivalent HPV vaccine at 0, 2, and 6 months. This study was conducted in compliance with regulations by an ethical committee review and was monitored by the Moffitt Cancer Center Protocol Support Office. The study population consisted of mid-adult aged males from two clinics located in Cuernavaca, Mexico and Tampa, Florida (the MAM Trial; www.clinicaltrials.gov, NCT01432574). A total of 145 men had specimens contributing to both month 0 and month 7 serum HPV antibody evaluations [7]. Here we evaluated anti-HPV-16 and anti-HPV-18 antibody avidity for 126 men. A subset of participants had peripheral blood mononuclear cells (PBMC) collected prior (N = 47) and post vaccination (N = 46), which were used for evaluating HPV-16 memory B-cell response.

#### 2.1.2. Vaccination and clinical procedure

The quadrivalent HPV vaccine, Gardasil (Merck & Co., Inc) containing HPV 6/11/16/18 virus-like particles (VLP) was used to vaccinate men at month 0, months 2 and 6 in a volume of 0.5 mL by intramuscular administration in the deltoid or thigh muscle. At month 0 and month 7 serum and PBMC specimens were collected and stored frozen at ‒80 °C until tested.

### 2.2. HPV ELISA

Specimens were tested for anti-HPV-16 and HPV-18 IgG levels by the L1 VLP an enzyme-linked immunosorbent assay (ELISA), as previously performed [8,18,19]. Flat-bottom high-binding microtiter plates (MaxiSorp, Nunc, Thermo Fisher Scientific, Waltham, MA) were coated with HPV-16 or HPV-18 L1 VLP at a concentration of 2.7 μg mL^‒1^ in 100 μl for at least 72 h at 4 °C. The plates were then washed three times with 350 μl of phosphate-buffered saline (PBS) containing 0.2% Tween 20 and blocked in 300 μl of PBS with 4% skim milk and 0.2% Tween-20 (blocking buffer) for 90 min at room temperature. The plates were washed three times. Serial dilutions of serum (starting dilution 1:100) in blocking buffer in 2-fold increments were added to the plate in a final volume of 100 μl. The plates were sealed and incubated at room temperature for 60 min on a plate shaker at 250 rpm. After washing the plate three times, peroxidase-labeled goat anti-human IgG (KPL, Inc.) was added and the plates were sealed and incubated at room temperature for 60 min on a plate shaker at 250 rpm. Plates were then developed with tetramethylbenzidine substrate solution (KPL, Inc.). After 25 min of incubation in the dark at room temperature, the reaction was stopped by the addition of 100 μl of 0.36 N H_2_SO_4_. The absorbance at 450 nm and 620 nm were measured with a microtiter plate reader (Spectramax M5; Molecular Devices, Sunnyvale, CA). Antibody levels were expressed as ELISA units (EU mL^‒1^) which were interpolated from the standard curve. Antibody levels were then normalized for total IgG in each specimen and expressed as EU mg^‒1^. Both the HPV-16 and HPV-18 ELISA were calibrated with the World Health Organizations International Standards. The International Units (IU) conversion factor for anti-HPV-16 antibodies is 1 IU mL^‒1^ = 5.29 EU mL^‒1^, and for anti-HPV-18 antibodies is 1 IU mL^‒1^ = 6.81 EU mL^‒1^.

### 2.3. Avidity assay

To approximate the binding strength of IgG antibodies to HPV, the avidity of antibodies was determined as previously described, using a modified ELISA-based method with the chaotropic agent guanidine hydrochloride (GuHCl; Sigma, St. Louis, MO) [20,21]. The avidity index is the concentration of GuHCl, expressed in Molar (M), that reduce the optical density by 50% compared to sample wells without GuHCl treatment. Flat-bottom microtiter plates were coated with HPV antigen, and blocked, as previously described above in the HPV ELISA method. Serum samples were diluted based on previous evaluation in the HPV-16 or HPV-18 ELISA to yield an absorbance reading of 1.0 ± 0.5. Samples were excluded if their reading was below 1.0 ± 0.5, or the sample could not be diluted down to 1.0 ± 0.5 with a 1:100 dilution. The diluted serum was incubated for 1 h at room temperature. Buffer alone, or 0.5 to 3.5 M GuHCl was added for 15 min at room temperature. After washing the plate, the secondary goat anti-human IgG was added for one hour at room temperature, and the plate was developed with TMB and the absorbance was read as previously described for the HPV ELISA.

### 2.4. Enzyme-linked immunosorbent spot (ELISpot) assay

Frozen PBMC were thawed, washed and resuspended in assay media containing RPMI 1640 (Gibco, Carlsbad, CA), 10% fetal bovine serum (HyClone, Logan, UT) and 1x penicillin–streptomy cin-glutamine (Gibco). Cell counts and viabilities were obtained with a MUSE Cell Analyzer (Millipore).

Approximately, 1 × 10^6^ viable cells per well were plated into 24-well tissue culture plates (Costar, Corning Inc.) in 2 mL stimulation media containing RPMI 1640 (Gibco), 10% fetal bovine serum (HyClone), 1x penicillin–streptomycin-glutamine (Gibco), 50 μM 2-mercaptoethanol (Sigma), 1 μM CpG ODN-2006 (Invitrogen), 1 μg mL^‒1^ Protein A from *Staphylococcus aureus* Cowan (Sigma), 1 μg mL^‒1^ pokeweed mitogen (Sigma). The stimulated PBMC were incubated in a 37 °C humidified 5% CO_2_ atmosphere for 6 days.

We adapted a previously established method for the HPV-16-specific memory B-cell ELISpot assay [22]. The day before assay setup, 96-well polyvinylidene fluoride (PVDF) membrane, HTS opaque plates (Millipore) were pre-wetted with 27% ethanol for < 1 min and washed four times with PBS (Lonza). HPV-16 VLP and negative control keyhole limpet hemocyanin (KLH) (Millipore) coating antigens were diluted to 5 μg per well and anti-IgG capture antibody (Mabtech) was diluted to 1.5 μg per well in PBS. The plates were then incubated overnight at 4 °C, washed four times with PBS and blocked with 200 μl per well with assay media for at least 30 min at room temperature. The 6-day stimulated PBMC were washed, counted and resuspended to plate 300,000 and 100,000 cells in the HPV-16 VLP and KLH wells and 10,000 cells in the total IgG wells. All conditions were plated in triplicate and incubated for 2 h at 37 °C and 5% CO_2_. After the two-hour incubation, the plates were washed six times with 0.05% Tween 20 (Sigma) in DPBS, followed by a 2-hour incubation at room temperature with a 1:500 dilution of biotinylated anti-IgG (Mabtech) in DPBS with 1% bovine serum albumin (Thermo Scientific) and 0.05% Tween 20. After incubation and four washes in DPBS to remove excess antibody, a 1:1000 dilution of streptavidin alkaline phosphatase (Mabtech) in DPBS with 1% bovine serum albumin (Thermo Scientific), was added to each well for 1 h at room temperature followed by 4 washes in DPBS. One hundred μl per well of BCIP/NBT substrate (KPL Inc., Gaithersburg, MD), was filtered and added for 7–10 min, resulting in the development of spots. The reaction was stopped by washing three times in distilled water. Plates were dried overnight, and the spots were visualized and counted using the ImmunoSpot Imaging Analyzer system (Cellular Technology Ltd.) and ImmunoSpot software v5.1. All wells were counted with set parameters and then each count was verified. The spot counts were adjusted to reflect the average number of spots per 1 × 10^6^ cells and then the frequency of antigen-specific B-cells was calculated by dividing the number of antigen-specific spots by the estimated number of total B-cells. Pre- and Post-vaccination timepoints were run simultaneously for each participant and a positive and negative donor control were run with each batch.

### 2.5. Statistical analysis

Geometric mean avidity responses were measured, and their associated 95% confidence intervals were calculated using GraphPad Prism Version 8.4.3. Statistical significance was determined using either a Mann-Whitney test or a Wilcoxon Matched pairs signed rank test between time points. For determining statistical significance between two clinical sites an unpaired *t*-test was used. Correlations between anti-HPV-specific IgG and anti-HPV-specific avidity indices were determined by Spearman correlation coefficients (ρ). Correlation between memory B-cell responses and avidity of anti-HPV-16 antibodies were determined by Spearman correlation coefficients (ρ). A *p* value of ≤ 0.05 was considered significant.

## 3. Results

### 3.1. Men who received three doses of the quadrivalent HPV vaccine had an increase in avidity for both HPV-16 and HPV-18

**Fig. 1.**
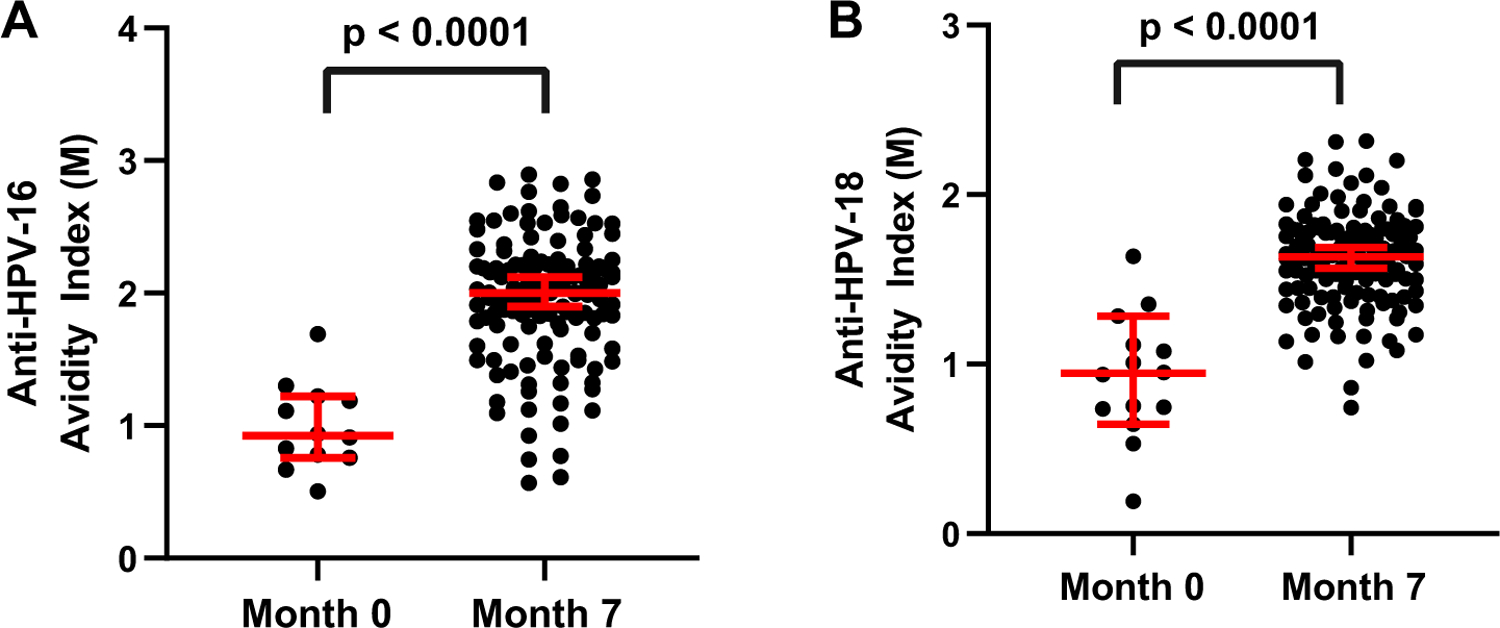
Avidity of HPV antibodies in serum pre and post vaccination with the quadrivalent HPV vaccine. A) Avidity of anti-HPV-16 antibodies in mid-adult aged men at month 0 before vaccination and month 7 post-dose three of vaccine. The avidity was measured in individuals with detectable HPV-16 antibody levels. B) Avidity of anti-HPV-18 antibodies in mid-adult aged men at month 0 before vaccination and month 7 post-dose three of vaccine. The avidity was measured in individuals with detectable HPV-18 antibody levels. The bars depict the 95% confidence intervals and the geometric mean. Statistical significance was calculated using a Mann-Whitney test between time points (p < 0.0001).

All men that received three doses of the quadrivalent HPV vaccine developed HPV-16 and HPV-18 antibodies [7]. The three doses of the quadrivalent vaccine also induced a significant increase in antibody avidity and thus, antibody affinity maturation at month 7 (Fig. 1). The avidity assay was not performed on the majority of month 0 samples because the antibody titers were below or close to the lowest detection levels. HPV vaccination induced an increase in HPV-16 antibody avidity from 0.9 M, at month 0 to 1.9 M at month 7 (Table 1). HPV-18 antibody avidity also increased from 0.8 M, at month 0 to 1.6 M at month 7 (Table 1). There was an overall 2-fold increase in both HPV-16 and HPV-18 avidity levels post vaccination.

**Fig. 2.**
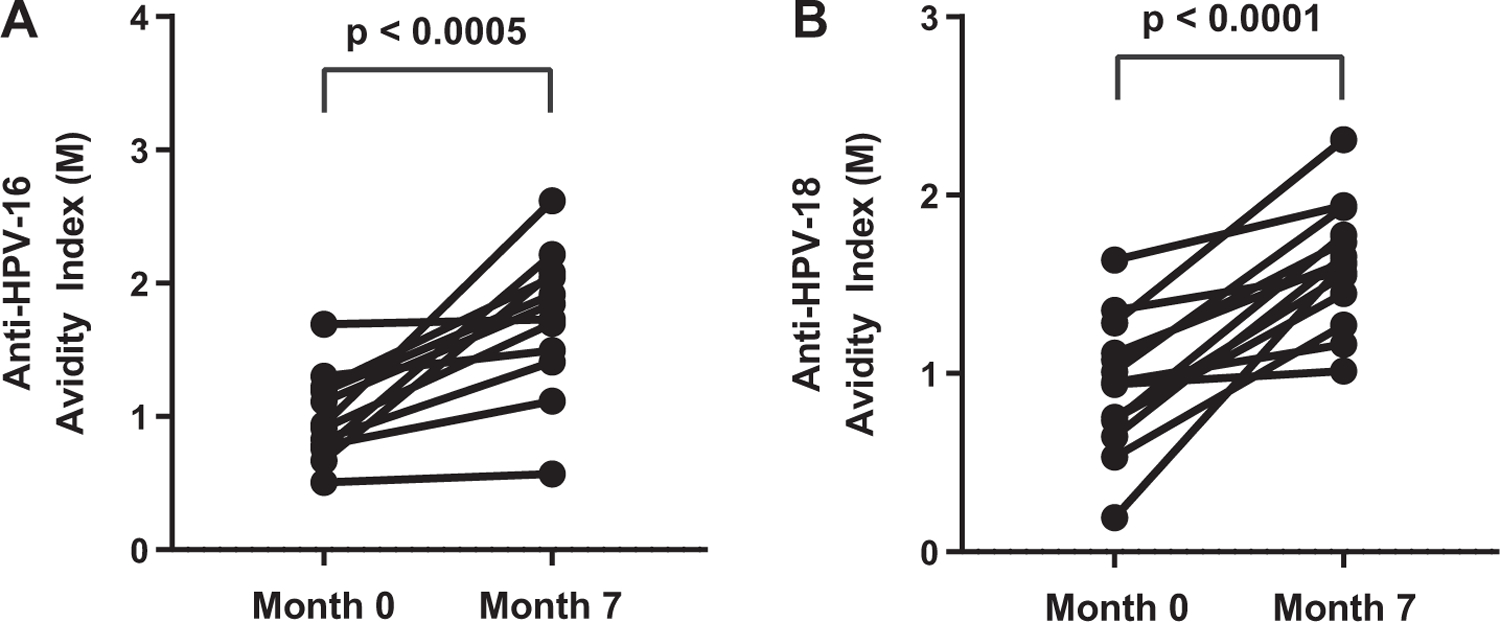
Paired analysis among measurable avidity levels at month 0 before vaccine versus month 7 post-dose three of vaccine. A) Anti-HPV-16 avidity index increased between month 0 and month 7. B) Anti-HPV-18 avidity index increased between month 0 and month 7. The circles represent individuals with a positive, detectable antibody response to HPV-16 or HPV-18, in which avidity can be measured. Statistical significance was determined using a Wilcoxon matched pairs signed rank test between time points.

Of the 150 participants, 24 were seropositive for HPV-16 and/or HPV-18 antibodies on day 1, and thus, had a previous history of infection with HPV-16 and/or HPV-18 before vaccination. Twenty-three seropositive participants also had an increase in antibody avidity after receiving three doses of vaccine (Fig. 2 and Table 2). There was an average increase in the anti-HPV-16 and anti-HPV-18 avidity index from month 0 to month 7 of 1.8 and 2.2, respectively. Of the 12 with a natural history of HPV-16 infection, only 3 individuals had a minimal increase of ≤ 1.2-fold in avidity (Fig. 2A). Of the 14 with a natural history of HPV-18 infection, 4 individuals had a minimal increase of ≤ 1.2-fold in avidity (Fig. 2B). The quadrivalent HPV vaccine increased avidity above what is seen with a natural history of infection with HPV.

In this study, the participants seropositive for HPV-16 differed by location, with a higher prevalence of positivity (13.3%) at month 0 in Mexico compared to 3.9% positivity in the US (Table 1). The participants seropositive for HPV-18 were similar with 11.8% and 10.7% positivity at month 0, for specimen from the US and Mexico, respectively (Table 1). After three doses of vaccine, all individuals mounted a robust immune response against HPV-16 and HPV-18. The avidity was also consistent across clinic locations for both HPV-16 and HPV-18; even though, their initial seropositivity differed. (Table 1).

### 3.2. Anti-HPV-18 avidity levels were moderately associated with the corresponding anti-HPV-18 antibody levels

**Fig. 3.**
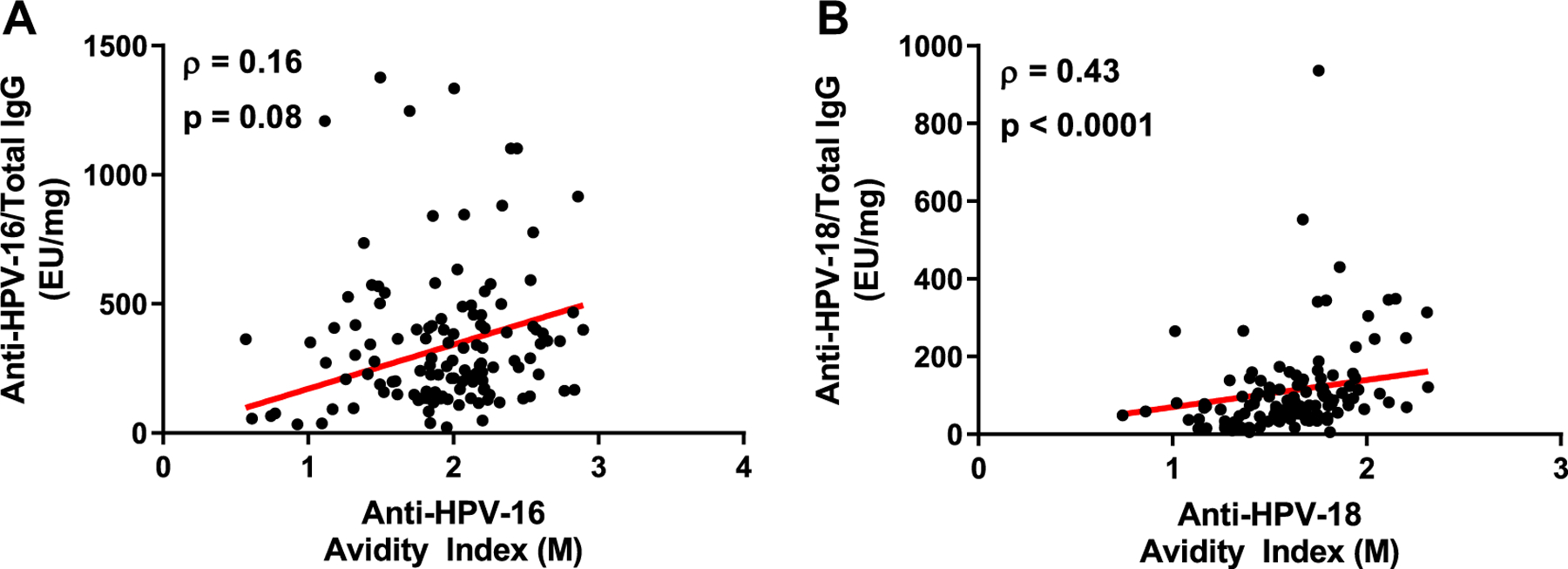
Correlation between total anti-HPV IgG antibodies and avidity at month 7 post vaccination. A) Anti-HPV-16 total IgG expressed as ELISA units per mg (EU mg^‒1^) from serum compared to avidity levels of anti-HPV-16 antibodies, B) Anti-HPV-18 total IgG expressed as ELISA units per mg (EU mg^‒1^) from serum compared to avidity levels of anti-HPV-18 antibodies. The Spearman correlation coefficient (ρ) and p-values are indicated on each graph.

The avidity levels of anti-HPV-16 antibodies post vaccination at month 7 did not correlate with the total anti-HPV-16 IgG levels (Fig. 3A). There were 11 individuals with high total anti-HPV-16 IgG levels (≥500 EU mg^‒1^) with avidity indices below the geometric mean and 63 individuals with avidity indices at or above the geometric mean with total anti-HPV-16 IgG levels below 500 EU per mg^‒1^. The concentration of circulating antibodies to HPV-16 does not appear to correlate with antibody affinity maturation for HPV-16. However, there was a moderate but significant correlation between anti-HPV-18 avidity levels and total HPV-18-specific IgG concentration (Spearman ρ = 0.43, p < 0.0001).

### 3.3. Memory B-cell responses was induced by the quadrivalent HPV vaccine

**Fig. 4.**
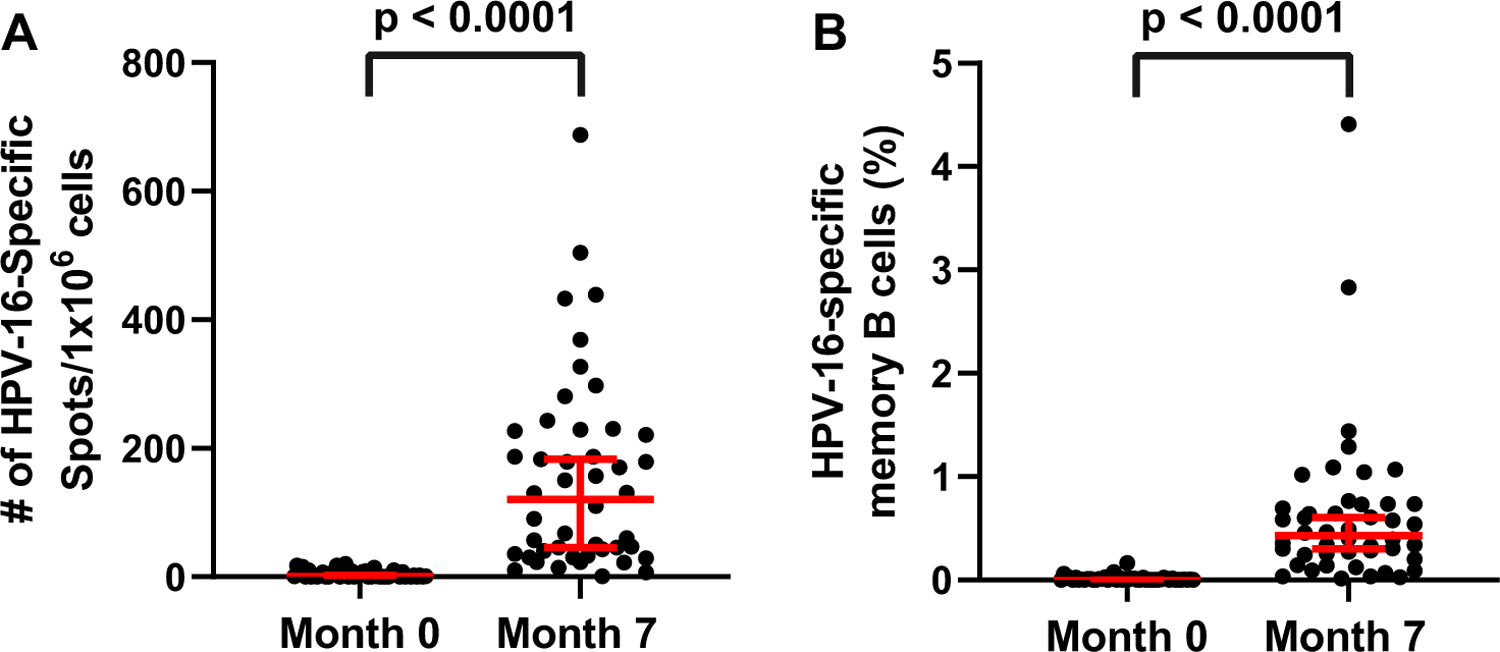
HPV-16-specific memory B-cell response pre and post vaccination with the quadrivalent HPV vaccine. A) Number of HPV-16-specific spots per 10^6^ cells in mid-adult aged men at month 0 before vaccination and month 7 post vaccination. B) The percent of HPV-16-specific memory B-cells in mid-adult aged men at month 0 before vaccination and month 7 post vaccination. The bars depict the 95% confidence intervals and the median. Statistical significance was calculated using a Mann-Whitney test between time points (p < 0.0001).

**Fig. 5.**
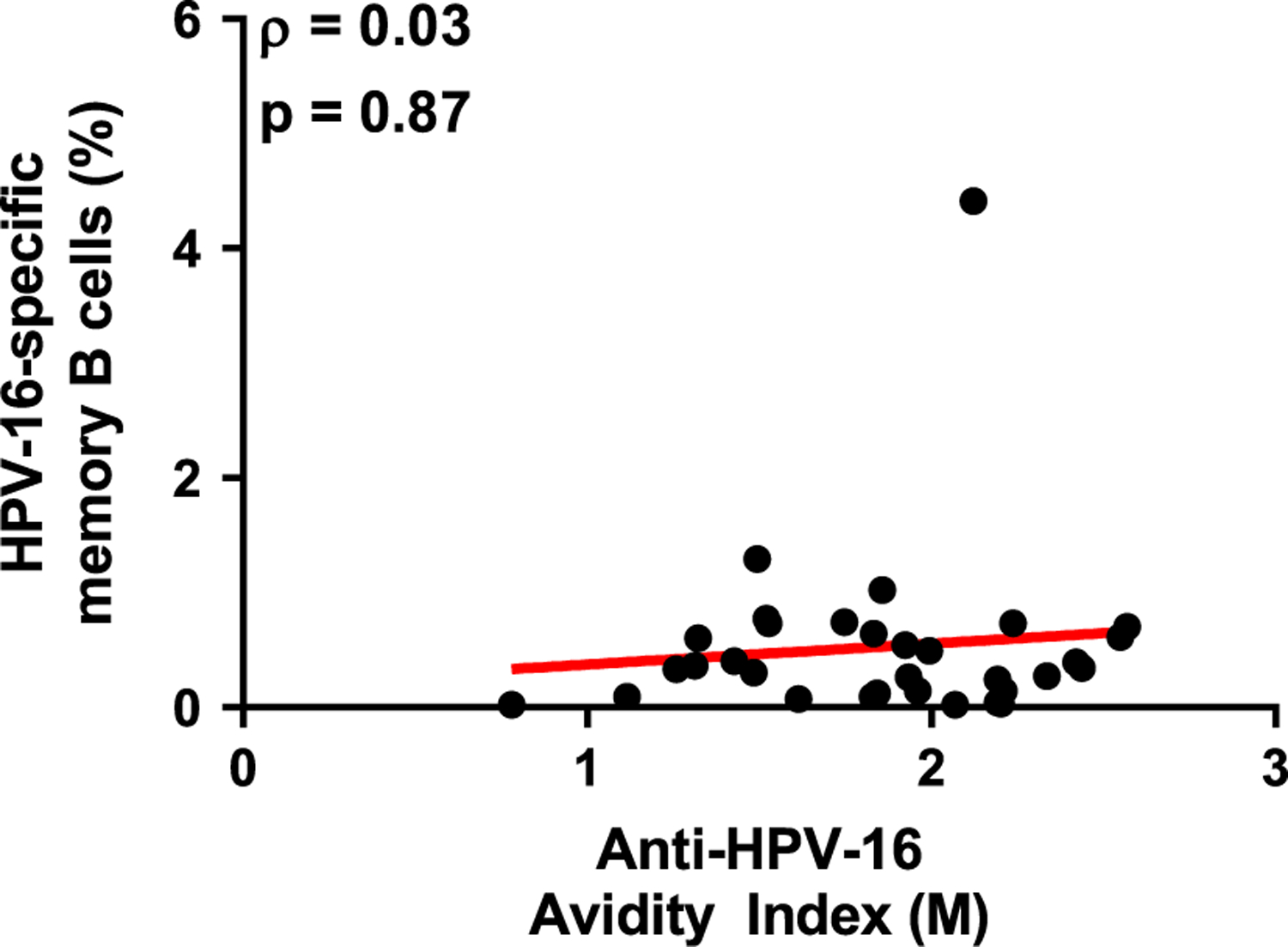
Correlation between memory B-cell responses and avidity of anti-HPV-16 antibodies. A) Correlation between the percent of HPV-16-specific memory B-cells and anti-HPV-16 avidity index (M). The Spearman correlation coefficient (ρ) and p-values are indicated on the graphs.

HPV-16 specific memory B-cells were enumerated from PBMC using the ELISpot assay. The antigen-specific memory B-cells were calculated as a percentage of the total IgG positive memory B-cells. Up to 4.4 % of IgG positive B-cells were HPV-16-specific post vaccination (Median = 0.43% of IgG positive B-cells) (Fig. 4A). Antigen-specific memory B-cells at month 0 were low (Median = 0.006% of IgG positive B-cells). There was a significant, 72-fold increase in the percent of memory B-cells that were HPV-16-specific between month 0 and month 7 (Table 3). However, there was no significant correlation between the frequency of anti-HPV-16-specific memory B-cells and the antibody avidity in men post-dose three of the quadrivalent HPV vaccine (Spearman ρ = 0.03, p = 0.87) (Fig. 5).

## 4. Discussion

Tracking antibody affinity and memory B-cells were two independent parameters to evaluate the humoral and B cellular immune response to HPV vaccination. The avidity assay measured the binding strength of antibodies to HPV and thus, an important immunogenicity marker. The ELISpot provided quantification of memory B-cells that were antigen specific and was another independent measurement for vaccine immunogenicity and potency. Three doses of the quadrivalent vaccine elicited antigen-specific memory B-cells similar to levels seen with the anthrax vaccine [23]. The frequency of HPV-16 specific memory B-cells did not appear to correlate with anti-HPV16 antibody avidity. In mice, repeat immunization significantly increased somatic mutation in GC not in memory B cells and had little effect on memory B cell antibody affinity [16]. There has been evidence of crossprotective vaccine efficacy of the bivalent HPV vaccine against HPV types that are not included in the vaccine, such as HPV-31, and this is associated with antibody responses [15]. The maintenance of systemic antibody levels is linked to memory B-cells, but this process did not appear to be correlated with antibody avidity for HPV-16. It would be interesting to determine if the antibody avidity for HPV-18 correlates with the frequency of antigen-specific memory B-cells, considering there was a modest correlation between the amount of anti-HPV-18 antibody and the antibody avidity to HPV-18. However, due to the limited number of PBMC we were unable to analyze HPV-18 memory B-cell responses in this study. Previous work showed no correlation between HPV-16 or HPV-18 specific avidity indices and the respective antibody titers for women at month 7 post vaccination with Cervarix [24].

Three doses of the quadrivalent HPV vaccine provided both a high quality and high quantity antibody response against HPV in mid-adult aged men. Vaccines against influenza and hepatitis B virus also showed similar induction of antibody quantity and quality. An increase in the concentration and avidity of influenza-specific IgG was also detected after three doses of influenza vaccine [25]. Protection afforded by the influenza vaccine was due to the quantity of vaccine-specific antibodies, and not antibody avidity or affinity [26]. An increase in the avidity of antibodies was observed after three doses of the hepatitis B vaccine, but did not correlate with the anti-hepatitis B IgG titers [27].

This was the first study to evaluate the memory B cell response in males after three doses of the quadrivalent HPV vaccine, and its relationship with avidity. In women that received the quadrivalent HPV vaccine there remained a high level of protection against cervical cancer even though anti-HPV antibody levels eventually dropped below detection, emphasizing the importance for immune memory [28]. Three doses of quadrivalent HPV vaccine increased antibody avidity and thus, affinity maturation for HPV-16/18 above the levels observed after natural infection prior to vaccination in mid-adult aged men. This vaccine regiment also increased the frequency of anti-HPV-16 memory B-cells, critical for immune memory and long-term protection against viral infection. The avidity of anti-HPV-16 antibodies does not correlate with the percent of HPV-16-specific memory B-cells. This work provides evidence that the ELISpot and avidity assay can be used to assess vaccine immunogenicity, and that these assays could be used to measure long term protection from HPV infections.

## Figures and Tables

**Table 1:** Avidity of anti-HPV-16 and HPV-18 antibodies prior and post vaccination in serum.

				Month 0		Month 7	
HPV type	Specimen Type	Clinic	N	AB positive (%)^a^	GMA^b^ (95% CI)	AB positive (%)^a^	GMA^b^ (95% CI)
16	Serum	U.S.	51	2 (3.9)	0.8 (0.6, 0.9)	51 (100)	1.9 (1.7, 2.0)
		Mexico	75	10 (13.3)	1.0 (0.8, 1.3)	75 (100)	1.9 (1.8, 2.0)
		Total	126	12 (9.5)	0.9 (0.8, 1.2)	126 (100)	1.9 (1.8, 2.0)
18	Serum	U.S.	51	6 (11.8)	0.6 (0.3, 1.0)	51 (100)	1.6 (1.5, 1.7)
		Mexico	75	8 (10.7)	1.1 (0.9, 1.4)	75 (100)	1.6 (1.5, 1.7)
		Total	126	14 (11.1)	0.8 (0.6, 1.1)	126 (100)	1.6 (1.5, 1.6)

a AB positive represents the number (%) of subjects whom met the criteria for measuring avidity of anti-HPV-16 and HPV-18 antibodies.

b GMA represents the geometric mean avidity response amongst subjects who met the criteria for measuring avidity of anti-HPV-16 and HPV-18 antibodies.

**Table 2:** Avidity levels in a subset of individuals with an increase in avidity between month 0 before vaccine versus month 7.

Participant	HPV-16 Avidity		HPV-18 Avidity	
	Month 0	Month 7	Month 0	Month 7
1	0.51	0.57	–	–
2	0.67	2.08	0.75	1.45
3	0.76	2.22	–	–
4	0.78	1.12	–	–
5	0.83	1.41	–	–
6	0.91	1.70	–	–
7	0.94	2.62	–	–
8	1.11	1.85	–	–
9	1.19	1.92	–	–
10	1.22	2.05	1.35	1.57
11	1.30	1.50	1.28	2.31
12	1.69	1.73	–	–
13	–	–	0.19	1.61
14	–	–	0.53	1.27
15	–	–	0.65	1.66
16	–	–	0.74	1.78
17	–	–	0.75	1.55
18	–	–	0.94	1.01
19	–	–	0.95	1.16
20	–	–	1.01	1.93
21	–	–	1.08	1.74
22	–	–	1.11	1.62
23	–	–	1.64	1.94

**Table 3:** HPV-16 memory B-cell responses prior and post vaccination in serum.

HPV type	Time	N	ELISpot (% of IgG specific) Median (95% CI)	N	ELISpot (# of Spots/10^6^ cells) Median (95% CI)
HPV-16	Month 0	47	0.006 (0–0.01)	47	2.2 (0–4.4)
	Month 7	46	0.43 (0.3–0.6)	46	120 (46–183)

a Median (middle value) and 95% CI of the median for percent HPV-16 specific-IgG memory B-cells.

b Median (middle value) and 95% CI of the median for number of HPV-16-specific spots per 10^6^ cells.

## Abbreviations:

AB: antibody
CI: confidence interval
ELISA: enzyme-linked immunosorbent assay
ELISpot: enzyme-linked immunosorbent spot
EU: ELISA units
GMA: geometric mean avidity
GuHCl: guanidine hydrochloride
HPV: human papillomavirus
IU: international units
M: molar
N: number
VLP: virus-like particle
